# Potential utility of risk stratification for multicancer screening with liquid biopsy tests

**DOI:** 10.1038/s41698-023-00377-w

**Published:** 2023-04-22

**Authors:** Elle S. Kim, Robert B. Scharpf, Montserrat Garcia-Closas, Kala Visvanathan, Victor E. Velculescu, Nilanjan Chatterjee

**Affiliations:** 1 Department of Biostatistics, Johns Hopkins Bloomberg School of Public Health, Baltimore, MD 21205 USA; 2 Department of Oncology, The Sidney Kimmel Comprehensive Cancer Center, Johns Hopkins University School of Medicine, Baltimore, MD 21205 USA; 3 Division of Cancer Epidemiology and Genetics, National Cancer Institute of Health, 9609 Medical Center Drive 7E-342, Rockville, MD 20850 USA; 4 Department of Epidemiology, Johns Hopkins Bloomberg School of Public Health, Baltimore, MD 21205 USA

**Keywords:** Cancer screening, Cancer genomics, Cancer models, Cancer prevention, Cancer epidemiology

## Abstract

Our proof-of-concept study reveals the potential of risk stratification by the combined effects of age, polygenic risk scores (PRS), and non-genetic risk factors in increasing the risk-benefit balance of rapidly emerging non-invasive multicancer early detection (MCED) liquid biopsy tests. We develop and validate sex-specific pan-cancer risk scores (PCRSs), defined by the combination of body mass index, smoking, family history of cancers, and cancer-specific polygenic risk scores (PRSs), to predict the absolute risk of developing at least one of the many common cancer types. We demonstrate the added value of PRSs in improving the predictive performance of the risk factors only model and project the positive and negative predictive values for two promising multicancer screening tests across risk strata defined by age and PCRS.

Developing effective screening tools for early cancer detection has long been a pressing interest due to the poor prognosis and survival associated with advancing cancer stage^1^. Identifying individuals at the subclinical or asymptomatic stage provides a unique window of opportunity for early intervention that has been shown to improve survival^2^. In a general population with a relatively low prevalence of cancer, ideally, a screening test needs to be broadly accessible, highly specific, and sensitive. A screening test should be specific to minimize overdiagnosis-related psychological and financial burdens and risks associated with unnecessary follow-up treatments and sensitive to prevent missed or interval cases. For these reasons, to date, the United States Preventive Service Tasks Forces has recommended only a handful of age-based single-cancer screening modalities such as colonoscopy^3^ (37.1% to 79.4% sensitivity (se) and 86.7% to 97.3% specificity (sp)) for colorectal cancer^4^ mammogram^5^ (86.9% se and 88.9% sp) for breast cancer^6^, low-dose computerized tomography^7^ (59% to 100% se and 26.4 to 99.7% sp) scan for lung cancer^8^, and pap test^9^ (70% to 80% se and 95% sp) for cervical cancer^10^.

Existing single-cancer screening tools face several challenges, including lack of adherence to screening recommendations^11–13^, low positive predictive value (PPV) or high false positives^14^, and missed or interval cancer cases^15^. Additionally, there are no presently accepted screening tools for many cancers with poor prognoses or high late-stage diagnosis rates for cancer detection in asymptomatic individuals. In this context, multicancer early detection (MCED) liquid biopsy tests using analytes such as cell-free DNA (cfDNA) are gaining traction^16–26^. Several recent studies have started to explore the feasibility of such approaches for early cancer detection in a limited clinical setting^18–28^. GRAIL’s Galleri test^21,26^ and Thrive’s DETECT-A^24^ (Detecting cancers Earlier Through Elective Mutation-based blood Collection and Testing) are of note.

MCED has the promise to lower cancer mortality, especially through early detection of cancers for which there is currently no screening available. However, as many recent studies have shown, the issue of low PPV persists as a significant limitation of the newly developed multicancer tests (Supplementary Table 1)^21,24,26,29^. The values of PPV for tests are anticipated to be highly influenced by specificity, and for a constant specificity value, by the combination of sensitivity and prevalence. The current MCED tests have high specificity (99% or higher) but typically have low PPV for the general population due to modest sensitivity and low prevalence of cancer in the general population. While sensitivity for some of these tests can be substantially higher for some specific cancers and may be improved further through the incorporation of additional features in cfDNA^19,27,28^, the PPV in most settings will still be expected to remain low as the prevalence of individual cancers is even lower. Thus, in the future, a risk-stratified approach is likely to be needed to enhance PPV and the risk-benefit balance of these tests. In the multicancer setting, however, population risk stratification becomes more challenging as one needs to consider risk factors across many cancers. There are also new opportunities due to the emergence of polygenic risk scores (PRSs) from genome-wide association studies (GWASs) across many cancers^30^. Recently, a study investigated the potential utility of PRSs and other risk factors to build a model for predicting risk for at least one of several cancers to understand the impact of lifestyle modifications on overall cancer risk^30^. However, the prospects of risk stratification in multicancer screening and the added values of PRS in addition to classical risk factors are yet to be investigated in the context of emerging MCED tests.

We use data from the prospective UK Biobank (UKBB) study and the US population cancer incidence rates to estimate future cancer risk given individuals’ genetic and nongenetic profiles. We identified the top ten incident cancer types for females and males with sufficiently large and publicly available GWAS (see Methods)^31^. Our final analysis involved 133,830 female and 115,207 unrelated male participants of White British ancestry aged 40–73, with 5807 and 5906 incident cancer cases for bladder, breast (female only), colorectum, endometrium, kidney, lung, melanoma, non-Hodgkin’s lymphoma, ovary, pancreas, and prostate (male only), respectively, over the course of follow-up. We used sex-specific Cox proportional hazards models where the baseline hazard is specified as a function of age and assumed the multiplicative effects of the risk factors^32^ with the outcome as the first cancer incidence of the above-mentioned cancers. PRSs were calculated for eleven (bladder, breast, colorectum, endometrium, kidney, lung, melanoma, non-Hodgkin’s lymphoma, ovary, pancreas, and prostate) cancer types (Supplementary Figs. 1 and 2). We included two major lifestyle-related exposures, namely smoking (status and pack-years of smoking) and body mass index (BMI), known to influence risk across multiple cancers, and family history of breast, colorectal, lung, and prostate cancer in non-adoptive first-degree relatives as risk factors. We then computed pan-cancer risk scores (PCRSs) as the weighted sum of the predictors included in the multicancer Cox model. The performance of the PCRSs was evaluated using a standardized hazard ratio for instantaneous risk and area under the curve (AUC) using up to 5 years of follow-up data. We assume that the probability of an individual carrying an asymptomatic, but screen-detectable cancer is proportional to the risk of incident cancer over a small-time interval (eleven months for DETECT-A and one year for Galleri). We use the Bayes theorem to determine expected PPVs and NPVs for DETECT-A and Galleri across the PCRS percentiles and ages based on reported diagnostic accuracies of these tests.

As anticipated, PCRSs were strongly associated with the risk of developing at least one cancer during the follow-up of the UKBB study in both females (HR: 1.39 per 1 SD, 95% CI: 1.33–1.45) and males (HR: 1.43 per 1 SD, 95% CI: 1.37–1.49) (Supplementary Table 2). We observed a strong degree of multicancer risk stratification by the combined effects of age, cancer-specific polygenic risk scores, and conventional risk factors shared across multiple cancer types (Figs. 1, 2, and Supplementary Figs. 3–6). Comparison of the 1-year and 10-year trajectories across various ages and risk strata for the PRS only model (Female AUC: 0.58, Male AUC: 0.59), risk factors only model (Female AUC: 0.55, Male AUC: 0.57), and the combined model (PCRS model; Female AUC: 0.60, Male AUC: 0.62), further demonstrates the added value of cancer-specific PRSs as covariates to improve multicancer risk stratification and predictive model performance (Supplementary Table 2 and Supplementary Figs. 3–6). The combined (i.e., PCRS) model showed a mean 1-year overall absolute cancer risk of 3.58% for the high-risk females aged 75 (top 10 percentile) and 0.77% for the low-risk females of the same age (bottom 10 percentile)—close to a 4.6-fold increase—whereas the risk factors only model showed a lower level of overall cancer risk stratification between the high-risk group and low-risk group, with 1-year absolute risks sitting at 2.36% and 1.12%, respectively, approximately corresponding to a 2.1-fold increase (Supplementary Fig. 3a–c).

**Fig. 1:**
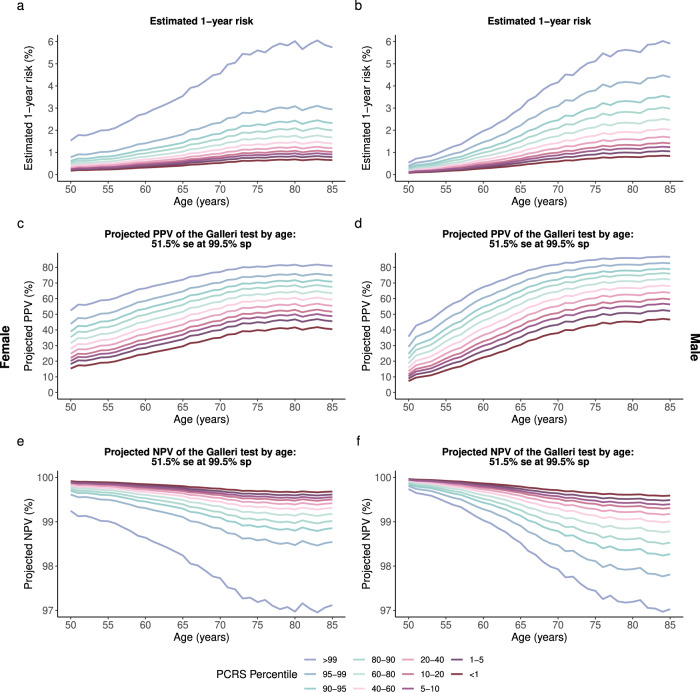
Estimated 1-year absolute risk and the projected PPV and NPV of the Galleri test with an overall sensitivity of 51.5% at 99.5% specificity for females and males. **a**, **b** Estimated 1-year risk of developing at least one of ten and eight cancer types for females and males, respectively. **c**, **d** Projected PPV of the Galleri test by age and PCRS percentile strata for females and males, respectively. **e**, **f** Projected NPV of the Galleri test by age and PCRS percentile strata for females and males, respectively. se sensitivity, sp specificity.

**Fig. 2:**
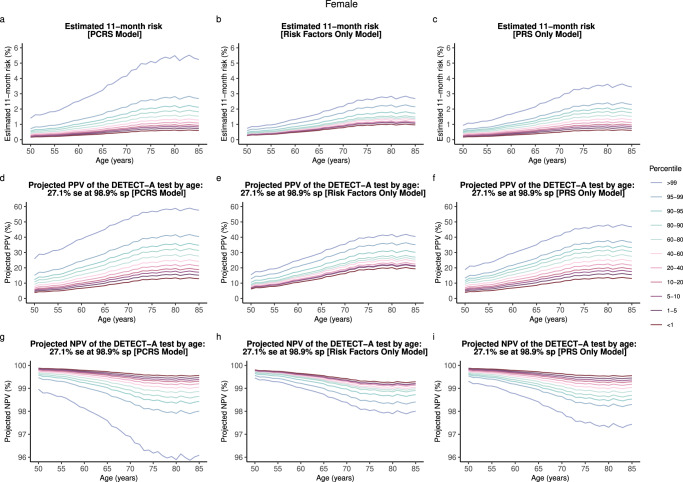
Comparison of the estimated absolute risk and the projected PPV and NPV of the DETECT-A test for three separate models (PCRS, Risk factors only, and PRS only). **a**–**c** Estimated 1-year absolute risk of developing one of the ten cancer types for the three separate multicancer risk prediction models. **d**–**f** Projected PPV of the DETECT-A test (27.1% sensitivity at 98.9% specificity) for three separate multicancer risk models. **g**–**i** Projected NPV of the DETECT-A test for three separate multicancer risk models. The pan-cancer risk score (PCRS) model uses cancer-specific PRSs and conventional risk factors (BMI, smoking status, pack-years of smoking, and family history of cancer in first-degree relatives) shared across multiple cancer type as covariates. Risk factors only model includes conventional risk factors as predictors. PRS only model includes the cancer-specific polygenic risk scores for bladder, breast, colorectum, endometrium, kidney, lung, melanoma, non-Hodgkin’s lymphoma (NHL), ovary, and pancreas as covariates. se sensitivity, sp specificity.

Further, the projected PPVs of the MCED tests (Galleri with a sensitivity of 51.5% at a specificity of 99.5% and DETECT-A with a sensitivity of 27.1% at 98.9% specificity) varied substantially by the level of the underlying risk of the population strata and the diagnostic accuracies of the liquid biopsy test in question (Figs. 1c, d, 2d–f). For example, 75-year-old females in the 90–95th PCRS percentile (AR: 2.27%) will have a 2.6-fold increased 1-year risk compared to the same-aged female in the 5–10th PCRS percentile (AR: 0.89%) (Fig. 1a). This corresponds to a PPV value of 70.3% and 48.2% for the Galleri test, translating to a 22.1% PPV difference for Galleri (Fig. 1c). NPV across all risk percentiles was reasonably high for both tests across all strata (Fig. 1e, f).

As the first step to integrating these new multicancer screening tests in the clinical setting, one can also consider a scenario in which a fixed threshold for PPV is employed as a metric to recommend early multicancer screening in the asymptomatic stage. The eligibility will strongly vary by both age and PCRS percentile. For example, at a threshold of 40% PPV, females could be eligible for the Galleri test as early as age 50 and in the 95th PCRS risk percentile and above (Fig. 1c). However, for DETECT-A, females would be eligible for screening starting at age 61 and in the highest PCRS risk percentile (Fig. 2d). Raising the PPV threshold to 60%, for DETECT-A, none of the females would achieve the required PPV even at the oldest age and highest risk groups (Fig. 2d). With Galleri’s high sensitivity and specificity, females will reach the desired threshold starting at age 56 and in the highest PCRS percentile (Fig. 1c).

Our analysis has several limitations. We assumed that the reported sensitivity and specificity of a test like DETECT-A and Galleri would be applicable across all age, sex, and risk groups. Given the increase in observed sequence alterations in cfDNA resulting from clonal hematopoiesis in older individuals^33–36^, improvements in cfDNA analyses will be needed to overcome these challenges, potentially through the use of mutation-agnostic methods^19,20,36^. Additional empirical data are needed to explore the potential heterogeneity of the diagnostic accuracy of MCED tests by age and other risk factors. In our multicancer risk prediction models, we included a limited set of risk factors, namely two major lifestyle-related factors that influence the risk of multiple cancers, family history of the most common cancers, and PRSs for each cancer type. We further assumed a proportional hazard model for all cancer risks, assuming multiplicative effects of age and all the other risk factors. Additional efforts are needed to build and validate more refined multicancer risk models in prospective cohort studies by including additional risk factors and interaction effects. Further, models^37,38^ that incorporate extensive family history information and carrier status for rare high-penetrant mutations would be important for individuals in high-risk families with strong clustering of related cancers. Excluding variants with minor allele frequency <0.01 is another limitation of our study.

We did not account for all cancer types in our multicancer model. To build a more robust and complete multicancer risk prediction model, cancer-specific risk models should first be developed and then combined to generate the risk of composite outcomes, like that of any cancer among several. The use of site-specific models will also allow PPV calculations to take into consideration underlying variations in the diagnostic accuracy of the MCED tests across different cancer types. Finally, our model-building effort was restricted to the participants of White British ancestry in the UKBB due to the limited sample size of other ancestry groups in this study, and also the lack of well-validated PRS in non-European ancestry populations. Large studies of diverse populations are urgently needed to study the accuracy of MCED tests and to build and validate robust multicancer risk prediction models across different racial and ethnic groups.

In summary, we conducted a first-of-a-kind study highlighting the potential for a risk-based approach in multicancer screening. We observed the added value of PRS in improving the degree of risk stratification for composite cancer outcomes compared to the model defined by age, family history, smoking, and BMI. In the context of population-level early cancer screening, the addition of PRS could allow the detection of high-risk individuals even in the absence of conventional risk factors. In the future, well-powered empirical studies are needed in diverse populations to prospectively evaluate the utility of the multicancer liquid biopsy tests for their use in personalized early cancer detection.

## Methods

Based on the report from the 2013–2017 United States Cancer Statistics (USCS) database, we identified the top ten malignant incident cancer types for females and males, after excluding non-melanoma skin cancer^31^. First, we surveyed the NHGRI-EBI Catalog of Published Genome-Wide Association Studies (GWAS Catalog)^39^ and the Polygenic Risk Score (PGS) Catalog^40^ to select the largest European ancestry-based GWAS as of May 2020 for each cancer type. We additionally browsed PubMed^41^ for large cancer-specific GWASs that were not included in the GWAS Catalog or PGS Catalog. For breast and colorectal cancer, we searched for prior European sample-based large-scale polygenic risk score (PRS) studies as of July 2020 and selected studies reporting the best-performing PRS (Supplementary Data). We did not consider pleiotropic GWAS. We filtered to cancer types with at least ten independent genome-wide significant SNPs after LD clumping at a genome-wide significant (GWS) *p*-value, 5E-8, threshold. Ultimately, eleven cancer types (bladder, breast, colorectum, endometrium, kidney, lung, melanoma, Non-Hodgkin’s lymphoma, ovary, pancreas, and prostate) were included in our analysis. For the full list of source literature and GWAS summary statistics included in our analysis, see Supplementary Data.

UK Biobank (UKBB) is a prospective epidemiological cohort study with over 500,000 participants^42–44^. Individuals aged 40–69 at baseline were recruited across the United Kingdom (UK) from 2006–2010^42–44^. A wide range of genotypic and phenotypic information, including personal medical and family history and lifestyle data, were collected at enrollment^42–44^. UKBB data is regularly updated by completing follow-up questionnaires, linkage to national cancer and mortality registries, and hospital inpatient electronic medical records systems^42–44^. With linkage to the national cancer registry data, cancer diagnosis date and type (coded based on International Classification of Disease 10 (ICD-10)) were available for participants diagnosed with cancer^42–44^. For our analysis, we used ICD-10 codes for cancer classification (see Supplementary Table 4).

We then filtered to unrelated UKBB participants of White British ancestry with imputed genotype data. We excluded individuals who were lost to follow-up, with genetic sex and self-reported sex mismatch, those with any cancer diagnosis prior to baseline assessment (prevalent cancers), and participants with missing data in any one of the classical risk factors (BMI, smoking status, pack years of smoking, and family history of cancer in non-adoptive first-degree relatives). In UKBB, family history of all cancers is not available. UK Biobank only reports family history of the top three cancer incident types for females (breast, bowel, and lung) and males (breast, bowel, and prostate). These quality control procedures resulted in a study population involving 133,830 females and 115,207 males.

After determining the source literature (Supplementary Data) for each cancer type, we reviewed the manuscript and any relevant additional resources. We extracted all autosomal SNPs from each cancer GWAS along with their summary statistics such as RSIDs, observed effect size estimates (OR or beta), effective (or risk) allele, risk allele frequency (RAF), and *p*-value. We excluded variants with minor allele frequency (MAF) < 0.01 and ambiguous SNPs (A/T or G/C allele) with MAF > 0.40. We filtered to variants with a MAF difference of less than 0.10 relative to the UK Biobank data. We removed variants with allele mismatches that could not be resolved by strand or dosage flips and/or SNPs with complete information mismatch, based on RSID, chromosome number, and position, to the European 1000 Genome reference panel^45^ or the UK Biobank data. We filtered to variants with an information score ≥0.90 based on the UK Biobank imputed genotype data. Finally, we used the fixed threshold approach to calculate PRS for each cancer. Using Plink^46^, we performed LD clumping at a *p*-value threshold of 5E-8, r^2^ of 0.1, and 1000 kb window with the European 1000 Genome reference panel^45^ as the reference panel to remove SNPs in linkage disequilibrium within each cancer type.

Then, PRS for UK Biobank participants was computed using PRSice2^47^.

The formula used for PRS calculation in PRSice2:

$PRS_j = \mathop {\sum }\limits_i^{} \beta _iSNP_{ij}$ where $PRS_j$ is the PRS for the jth individual, *β*_*i*_ is the observed effect size estimate for the ith SNP, and $SNP_{ij}$ is the dosage information for the effective allele of the ith SNP for the jth individual. We standardized each PRS to have unit variance and zero mean.

We developed a sex-specific pan-cancer risk prediction model to estimate the risk of developing at least one cancer over the course of follow-up. The multicancer model included eleven cancer types (bladder, breast [Female only], colorectum, endometrium [Female only], kidney, lung, melanoma, Non-Hodgkin’s lymphoma, ovary [Female only], pancreas, and prostate [Male only]). Data were split into 2/3 training set and 1/3 of test set—independent validation datasets used for model performance evaluation and subsequent analysis.

Cox proportional hazard regression (Cox) model^32^ was fitted to the training set with the outcome as an incidence of any first cancer included in the analysis. The models specified a baseline hazard as a function of age and assumed multiplicative effects of the risk factors^32^:

$$\lambda \left( {t|{{{\boldsymbol{z}}}}} \right) = \lambda _0(t)\exp \left( {\beta _1z_1 + \beta _2z_2 + \ldots + \beta _nz_n} \right)$$

t: time-to-event; time to any first cancer incidence, censoring age, or death age

$\lambda _0(t)$: baseline hazard function

***z*** = (*z*_1,_ *z*_2, …,_ *z*_*n*_): set of covariates (risk factors) included in the Cox model

***β*** = (*β*_1_, *β*_2_, …, *β*_*n*_): set of coefficients (log hazard ratios) for the predictors

Polygenic risk scores for each cancer (Supplementary Figs. 1 and 2), family history of cancer (breast, colorectum, lung, and prostate) in any first-degree relatives (nonadopted), body mass index, and pack-years of smoking were included as predictors in the model. We also adjusted for the first ten principal components. Also, as UKBB is a left-truncated and right-censored cohort, we used age as the timescale for the Cox model—that is, participants enter the model at recruitment age and exit at cancer incidence age, censoring age, or death age–whichever occurs first. We used the censoring date for the cancer registry data provided by UKBB^48^. In the underlying analysis of the UK Biobank data using the Cox proportional hazard model, the “event” is defined as the occurrence of any of these cancers, and the “time-to-event” is the time to first onset of any of these cancers. Thus, if an individual has multiple cancers, e.g., lung cancer first and then prostate, the individual is censored at the onset of the lung cancer. Further, if an individual first develops cancer of a type other than the ones included in our list, then they are censored at the first onset of those cancer types. Further, deaths from non-cancer causes were also treated as censoring events. Thus, the underlying hazard ratio parameters of the model can be interpreted as the instantaneous risk of developing at least one among the set of selected cancers, given a person was free of *all cancers* up to that time point.

Additionally, recognizing the concerns with the imputation of clinical/epidemiologic data, we conducted a complete-case analysis for the paper. A total of ~19% of subjects were removed who have missing data in any of the risk factors. Pack-years of smoking had the highest amount of missing data (~16%) missing, but all other individual variables had a small missing rate (<5%). For demonstrating the risk-stratification ability of models, a complete-case analysis is more desirable as imputation and model averaging will cause a diminishing of risk-stratification compared to the full potential of the model. In other words, our goal is to demonstrate the risk-stratification ability of the models for a population in which the underlying risk factors could be fully observed. From that point of view, a complete-case analysis is more desirable.

We computed pan-cancer risk scores (PCRS) or cancer-specific risk scores for all UKBB participants as the weighted sum of the predictors, with weights for each predictor as the estimated log hazard ratio (HRs) from the fitted Cox model. Then, in the test set, we assessed the discriminatory accuracy of the pan-cancer risk score (PCRS) or the cancer-specific risk score (for individual cancer models) using Harrel’s concordance index (C-statistic) and area under the curve (AUC) at five years of follow-up.

We used iCARE (Individualized Coherent Absolute Risk Estimation)^49^ to estimate absolute risk. Detailed methodology for absolute risk model building is described in Choudhury et al. 2020^49^. Briefly, risk estimates for each individual in the test set were obtained by feeding age-specific cancer incidence rates by 1-year strata, log HR parameters from the Cox model, and the reference dataset into the model. We used 2016 cancer incidence rates in white individuals of the SEER*Stat database^50^. Site-specific cancer incidence rates were obtained and then added to get the overall incidence rates for any cancer included in our study. Cancer incidence rates for a given age and sex were determined by the following year’s cancer incidence rates. For instance, in our study, cancer incidence rates for females aged 50–51 will correspond to SEER*Stat’s cancer incidence rates for females aged 51–52. This is to account for the fact that the DETECT-A test was performed at study enrollment, and the female participants were followed up over the course of 12 months. DETECT-A and Galleri will both be used to detect cancers early, prior to conventional diagnosis. The reference dataset was obtained by simulating 10,000 samples representative of the underlying UKBB population using the normal distribution with PCRS or cancer-specific risk score mean and standard deviation.

DETECT-A study reported an overall sensitivity of 27.1% at 98.9% specificity and an empirical PPV value of 19.4% (95% CI: 13.1–27.1%)^24^. We wanted to select a time window for absolute risk estimation so that the PPV for females aged 65–75 is equal to the point estimate of 19.4% reported in the DETECT-A study^24^. We varied the time window by one month around one year and calculated the weighted average PPV for females aged 65–75 based on the UKBB PCRS distribution and age distribution as reported by the US Census Bureau^51^. We found that a time window of 11 months provided the best match for the overall PPV for the 65–75 group to the empirically determined PPV value of 19.4%. Thus, subsequently, we calculated PPV and NPV for different age and PCRS risk groups based on underlying 11-month absolute risk.

Galleri reported an overall sensitivity of 51.5% at 99.5% specificity. For Galleri, we used a time window of 1-year^21,26^. For DETECT-A, we omit the calculation of projected PPVs and NPVs for males as it does not include prostate cancer (highest incident cancer for males) as one of the detectable cancer types^50^.

Given the absolute risk estimate, *x*, the positive predictive value and negative predictive value of the multicancer liquid biopsy test can be calculated using the formula below:

$$Se = sensitivity;Sp = specificity$$

$$PPV(x) = \frac{{Se \times p(x)}}{{Se \times p(x) + \left( {1 - Sp} \right) \times \left( {1 - p(x)} \right)}}$$

$$NPV(x) = \frac{{Sp \times (1 - p(x))}}{{\left( {1 - Se} \right) \times p(x) + Sp \times \left( {1 - p(x)} \right)}}$$

The absolute risk estimate can be written as a function of age and risk factors. We assumed that the sensitivity and specificity of the multicancer liquid biopsy test do not depend on the underlying risk factors, and we used the value of these as reported from the DETECT-A and Galleri study (Supplementary Table 1)^24,26^.

This study was conducted under UK Biobank Application Number 17712 (PI: Dr. Nilanjan Chatterjee). The study analyzes existing UK Biobank data and does not involve new human research participants. UK Biobank was approved by the North West Multi-center Research Ethics Committee (https://www.ukbiobank.ac.uk/learn-more-about-uk-biobank/about-us/ethics).

### Reporting summary

Further information on research design is available in the Nature Research Reporting Summary linked to this article.

## Supplementary information

Supplementary Data

Supplementary Information

REPORTING SUMMARY

## Data Availability

UK Biobank data are available through an application to the UK Biobank Access Management System (AMS), https://www.ukbiobank.ac.uk/enable-your-research/register. GWAS summary statistics of the single-nucleotide polymorphisms (SNPs) used for polygenic risk score (PRS) construction for each trait is available in the Supplementary Data section with relevant source literature.

## Code availability

This project was developed using R version 4.2.2. and the codes used for the project are available from Github at https://github.com/eswk-im/PCRSanalysis-.git.
