# Supplementary material for: Potential utility of risk stratification for multicancer screening with liquid biopsy tests
Source: NPJ Precis Oncol. 2023 Apr 22;7:39. doi: 10.1038/s41698-023-00377-w (PMC10122653; doi:10.1038/s41698-023-00377-w)
Supplement: Supplementary file 2 — Supplementary Information [file 41698_2023_377_MOESM2_ESM.pdf]

Supplementary Tables and Figures

Female – HR per SD

PRS	HR per SD (95% CI)	Number of Cases	P
Bladder	1.41 (1.09–1.83)	57	8.92e–03
Breast	1.58 (1.53–1.64)	2855	2.15e–133
Colorectum	1.49 (1.39–1.61)	681	7.08e–26
Endometrium	1.25 (1.14–1.36)	477	1.30e–06
Kidney	1.22 (1.02–1.45)	127	2.77e–02
Lung	1.18 (1.08–1.28)	494	1.23e–04
Melanoma	1.78 (1.62–1.96)	408	5.17e–33
Non–Hodgkin's Lymphoma	1.16 (1.03–1.30)	277	1.42e–02
Ovary	1.16 (1.03–1.31)	282	1.33e–02
Pancreas	1.37 (1.16–1.61)	149	1.43e–04

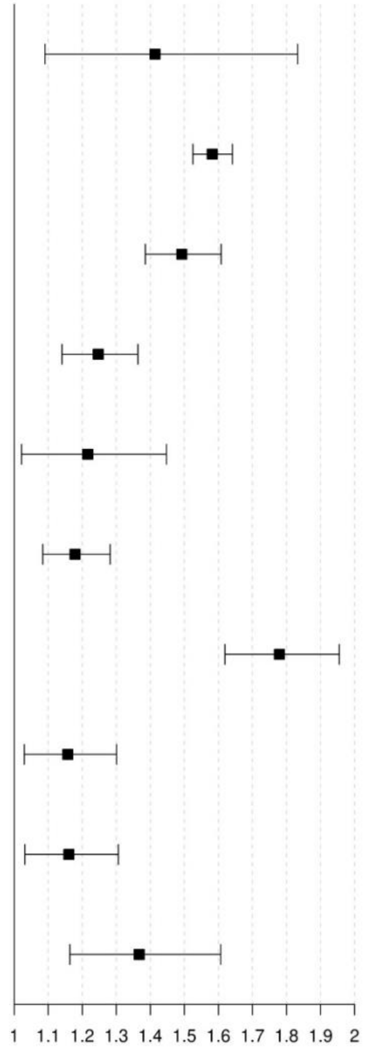

Supplementary Figure 1. The estimated hazard ratios (HR) per standard deviation (SD) of polygenic risk score (PRS) by cancer type [Female]. For each cancer type, Cox proportional hazard (Cox) model was fit to the female UK Biobank (UKBB) cohort to evaluate the association between time to cancer incidence and cancer-specific PRS. We adjusted for the first ten genetic principal components. Each model uses age as a timescale. Individuals who were lost to follow-up, with genetic sex and self-reported sex mismatch and those with prevalent cancers were excluded from the analysis. Each PRS is Z-score normalized to mean (μ) of 0 and standard deviation (σ) of 1. CI: Confidence Interval; HR: Hazard Ratio; SD: Standard Deviation; P: P-value; PRS: Polygenic Risk Score.

Male – HR per SD

PRS	HR per SD (95% CI)	Number of Cases	P
Bladder	1.28 (1.12–1.46)	230	1.91e–04
Colorectum	1.51 (1.42–1.61)	957	1.91e–37
Kidney	1.25 (1.10–1.41)	249	4.35e–04
Lung	1.20 (1.11–1.30)	554	6.57e–06
Melanoma	1.71 (1.55–1.89)	386	1.49e–27
Non–Hodgkin's Lymphoma	1.22 (1.10–1.36)	326	2.76e–04
Pancreas	1.39 (1.20–1.61)	182	8.98e–06
Prostate	1.78 (1.72–1.85)	3022	6.55e–238

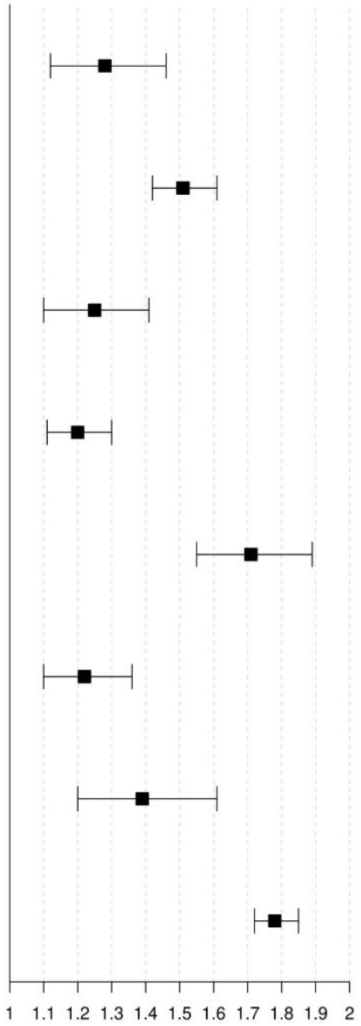

Supplementary Figure 2. The estimated hazard ratios (HR) per standard deviation (SD) of polygenic risk score (PRS) by cancer type [Male]. For each cancer type, Cox proportional hazard (Cox) model was fit to the male UK Biobank (UKBB) cohort, respectively, to evaluate the association between time to cancer incidence and cancer-specific PRS. We adjusted for the first ten genetic principal components. Each model uses age as a timescale. Individuals who were lost to follow-up, with genetic sex and self-reported sex mismatch or those with prevalent cancers were excluded from the analysis. Each PRS is Z-score normalized to a mean (μ) of 0 and standard deviation (σ) of 1. CI: Confidence Interval; HR: Hazard Ratio; SD: Standard Deviation; P: P-value; PRS: Polygenic Risk Score.

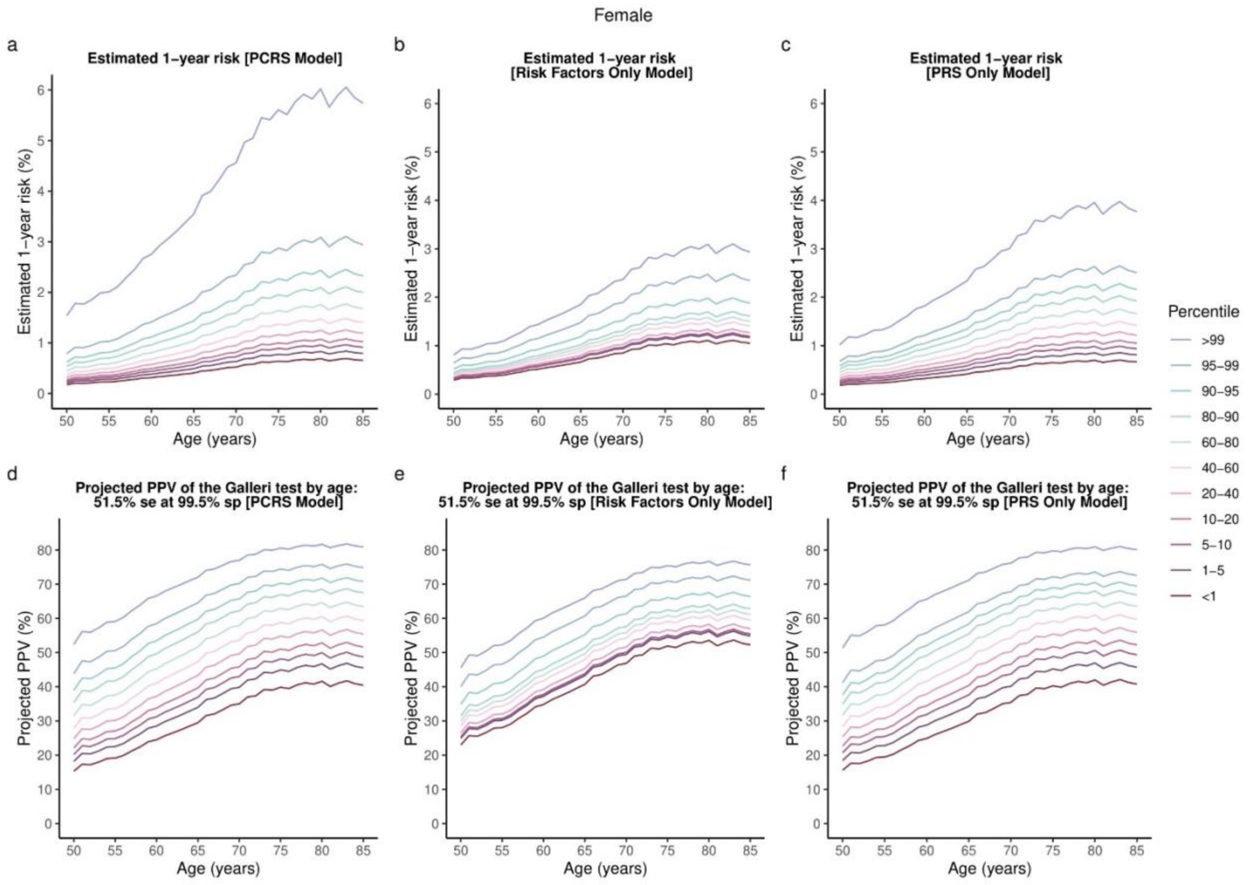

Supplementary Figure 3. Comparison of the 1-year absolute risk and the projected PPV of the Galleri test for the PCRS, Risk factors only, and PRS only models [Female]. a, b, c) Estimated 1-year absolute risk of developing one of the ten cancer types for the three separate multicancer risk prediction models. d, e, f) The corresponding PPV of the Galleri test (51.5% se, 99.5% sp) for three separate multicancer risk models. The pan-cancer risk score (PCRS) model uses cancer-specific PRSs and conventional risk factors (BMI, smoking status, pack-years of smoking, and family history of cancer in first-degree relatives) shared across multiple cancer types as covariates. Risk factors only model includes conventional risk factors as predictors. The PRS only model includes the cancer-specific polygenic risk scores for bladder, breast, colorectum, endometrium, kidney, lung, melanoma, non-Hodgkin's lymphoma (NHL), ovary, and pancreas as covariates. se: sensitivity; sp: specificity

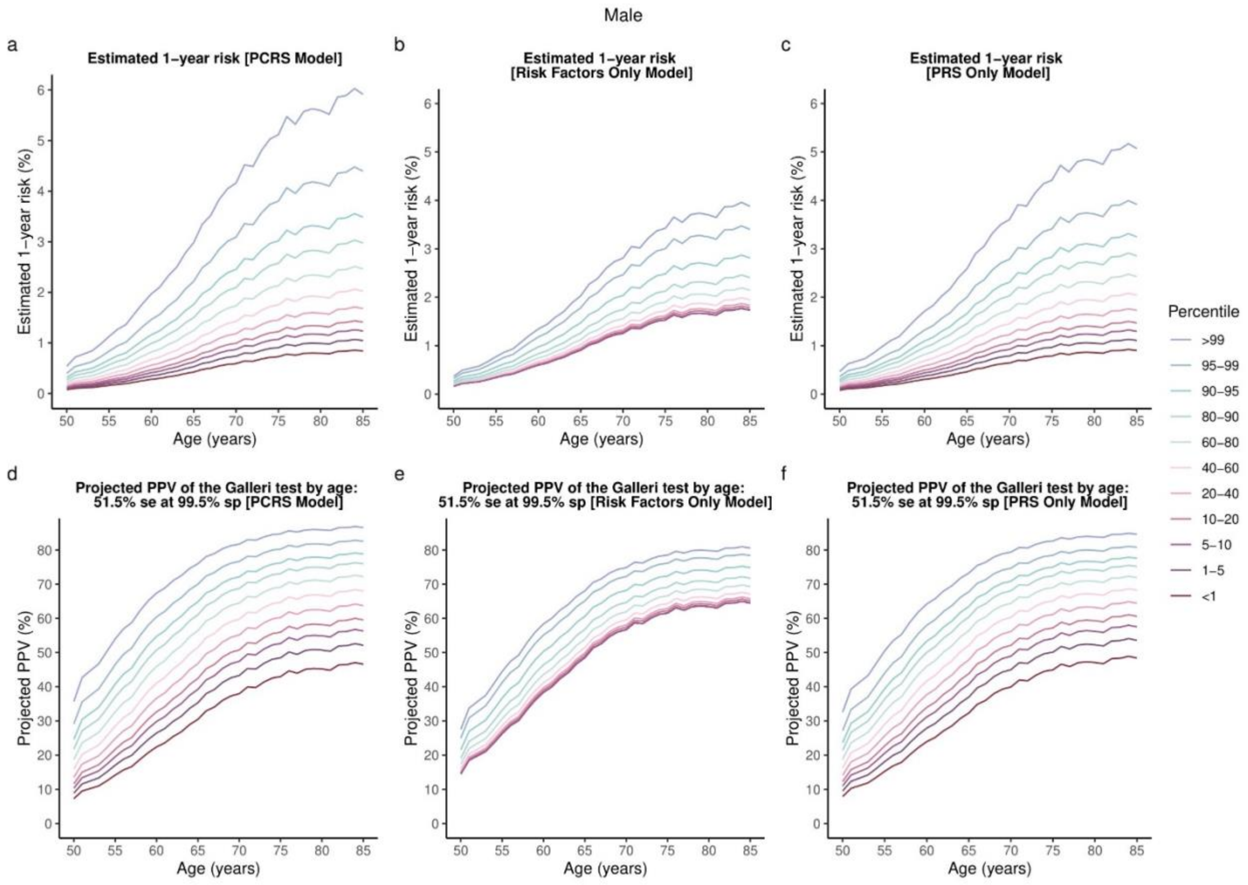

Supplementary Figure 4. Comparison of the 1-year absolute risk and the projected PPV of the Galleri test for the PCRS, Risk factors only, and PRS only models [Male]. a, b, c) Estimated 1-year absolute risk of developing one of the ten cancer types for the three separate multicancer risk prediction models. d, e, f) The corresponding PPV of the Galleri test (51.5% se, 99.5% sp) for three separate multicancer risk models. The pan-cancer risk score (PCRS) model uses cancer-specific PRSs and conventional risk factors (BMI, smoking status, pack-years of smoking, and family history of cancer in first-degree relatives) shared across multiple cancer types as covariates. Risk factors only model includes conventional risk factors as predictors. The PRS only model includes the cancer-specific polygenic risk scores for bladder, colorectum, kidney, lung, melanoma, non-Hodgkin's lymphoma (NHL), pancreas, and prostate as covariates. se: sensitivity; sp: specificity

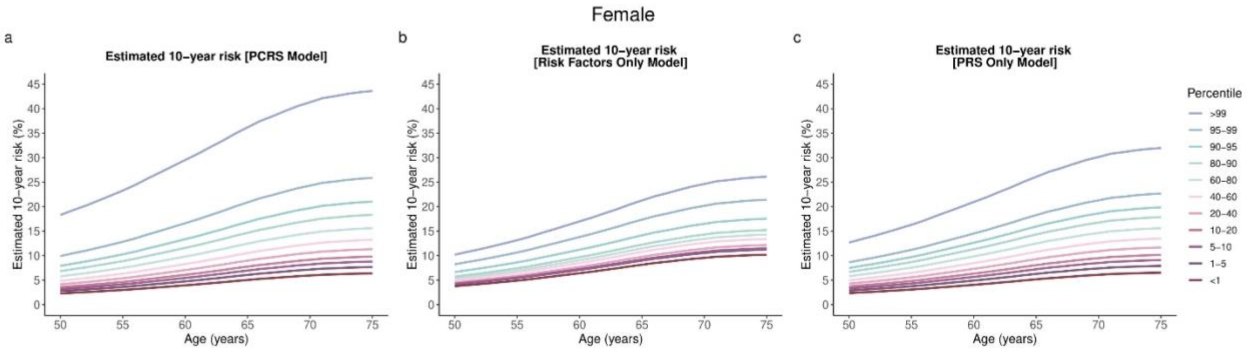

Supplementary Figure 5. Comparison of the estimated 10-year risk for the PCRS, Risk factors only, and PRS only models [Female] a, b, c) Estimated 10-year absolute risk of developing one of the ten cancer types for the three separate multicancer risk prediction models. The pan-cancer risk score (PCRS) model uses cancer-specific PRSs and conventional risk factors (BMI, smoking status, pack-years of smoking, and family history of cancer in first-degree relatives) shared across multiple cancer types as covariates. Risk factors only model includes conventional risk factors as predictors. The PRS only model includes the cancer-specific polygenic risk scores for bladder, breast, colorectum, endometrium, kidney, lung, melanoma, non-Hodgkin's lymphoma (NHL), ovary, and pancreas as covariates. se: sensitivity; sp: specificity

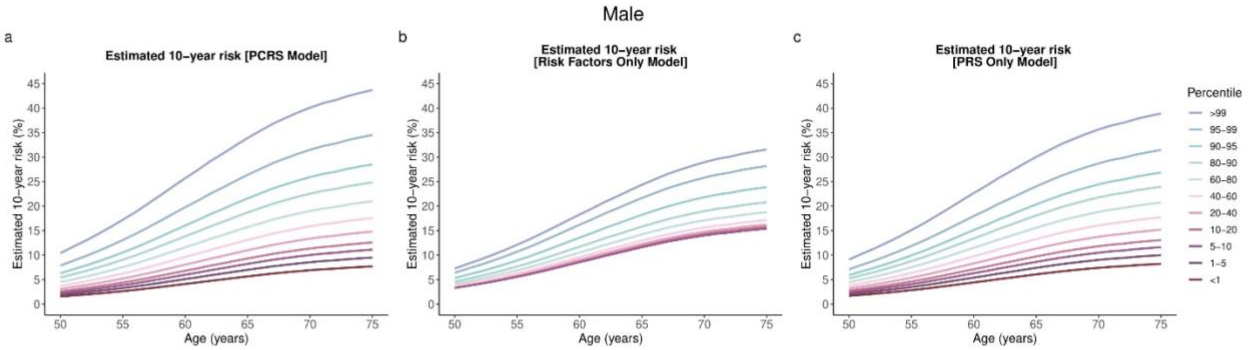

Supplementary Figure 6. Comparison of the estimated 10-year risk for the PCRS, Risk factors only, and PRS only models [Male] a, b, c) Estimated 10-year absolute risk of developing one of the eight cancer types for the three separate multicancer risk prediction models for males. The pan-cancer risk score (PCRS) model uses cancer-specific PRSs and conventional risk factors (BMI, smoking status, pack-years of smoking, and family history of cancer in first-degree relatives) shared across multiple cancer types as covariates. Risk factors only model includes conventional risk factors as predictors.The PRS only model includes the cancer-specific polygenic risk scores for bladder, colorectum, kidney, lung, melanoma, non-Hodgkin's lymphoma (NHL), pancreas, and prostate as covariates. se: sensitivity; sp: specificity

DETECT-A

DETECT-A	All Cancers
Stage I-IV Sensitivity at 98.9% Specificity	27.1%
1-Year PPV of DETECT-A (Female-only)	19.4%

Galleri

Galleri	All Cancers
Stage I-IV Sensitivity at 99.5% Specificity	51.5%
1-Year Projected PPV of Galleri (Female and Male Combined)	44%

Supplementary Table 1. The reported performance of the DETECT-A and Galleri blood tests [1,2]. DETECT-A: Detecting cancers Earlier Through Elective mutation-based blood Collection and Testing.

Female, Multicancer Model N = 44,610, number of events = 1,936			
[a]PRS$_{bladder}$ + … + PRS$_{pancreas}$ + [b]Risk Factors			
**HR per SD (95% CI)**	**P**	**5 Year AUC**	**C-Statistics (SE)**
1.39 (1.33 – 1.45)	4.35E-54	0.60	0.60 (0.007)
[b]Risk Factors Only			
**HR per SD (95% CI)**	**P**	**5 Year AUC**	**C-Statistics (SE)**
1.20 (1.15 – 1.25)	1.62E-20	0.55	0.55 (0.007)
[a]PRS$_{bladder}$ + … + PRS$_{pancreas}$			
**HR per SD (95% CI)**	**P**	**5 Year AUC**	**C-Statistics (SE)**
1.33 (1.27 – 1.39)	5.47E-36	0.58	0.58 (0.007)
**Male, Multicancer Model N = 38,403, number of events = 1,969**			
[c]PRS$_{bladder}$ + … + PRS$_{prostate}$+ [b]Risk Factors			
**HR per SD (95% CI)**	**P**	**5 Year AUC**	**C-Statistics (SE)**
1.43 (1.37 – 1.49)	6.10E-61	0.62	0.61 (0.007)
[b]Risk Factors Only			
**HR per SD (95% CI)**	**P**	**5 Year AUC**	**C-Statistics (SE)**
1.18 (1.13 – 1.22)	1.02E-16	0.57	0.55 (0.007)
[c]PRS$_{bladder}$ + … + PRS$_{prostate}$			
**HR per SD (95% CI)**	**P**	**5 Year AUC**	**C-Statistics (SE)**
1.37 (1.31 – 1.43)	1.72E-45	0.59	0.59 (0.007)

[a]Bladder, breast, colorectum, endometrium, kidney, lung, melanoma, non-Hodgkin's lymphoma (NHL), ovary, pancreas PRS
[b]Body mass index (BMI), smoking status, pack years of smoking, family history of breast, colorectal, lung, and prostate cancer in a first-degree relatives (nonadopted father, mother, and siblings)
[c]Bladder, colorectum, kidney, lung, melanoma, non-Hodgkin's lymphoma (NHL), pancreas, prostate PRS

Supplementary Table 2. Comparison of performance of the Pan-cancer risk score (PCRS) model, Risk factors only model, and PRS only model in the test set. The linear predictor for each model is estimated as the weighted sum of the covariates included in each model. The weights are hazard ratios computed by fitting the Cox model to the training data. The performance of linear predictor was evaluated in the test set. Hazard ratios (HR) per SD, the corresponding p-value, Harrell's C-index, and the area under the curve (AUC) is reported. AUC: area under the curve; CI: Confidence Interval; C-statistic: Harrell's C-index; HR: Hazard Ratio; SD: Standard Deviation; P: p-value.

		Female		Male	
**Parameter**	**Groups**	**Person-years (full female cohort)**	**Person-Years (Incident Cancer Cases Cohort)**	**Person-years (full male cohort)**	**Person-Years (Incident Cancer Cases Cohort)**
**Age at Study Entry**	**<44**	81178	1008	75836	232
	**45-49**	126204	1720	106832	809
	**50-54**	150195	2668	121314	1903
	**55-59**	173338	4290	140745	3564
	**60-64**	227500	6235	189102	7850
	**65+**	175865	5659	163200	8474
**Body Mass Index**	**Normal (18.5 kg/m^2 - 24.9 kg/m^2)**	367453	7189	202515	5397
	**Underweight (< 18.5 kg/m^2)**	6935	127	1664	62
	**Overweight (25 kg/m^2 - 29.9 kg/m^2)**	342855	8298	393678	11720
	**Obese (> 30 kg/m^2)**	217069	5967	199179	5653
**Family History of Cancer**	**Yes**	344168	8885	291227	9713
	**No**	590144	12696	505808	13119
**Smoking Status & Pack Years of Smoking**	**Never Smoker**	658987	14065	482250	11726
	**Former Smoker with Heavy Pack Years of Smoking (> 40 pack-years of smoking)**	16398	732	39491	2076
	**Former Smoker with Moderate Pack Years of Smoking (20 - 40 pack years of smoking)**	56695	1740	76394	2640
	**Former Smoker with Light Pack Years of Smoking (0 - 20 pack years of smoking)**	133512	2940	124000	3641
	**Current Smoker with Heavy Pack Years of Smoking (> 40 pack years of smoking)**	11188	636	22737	1282
	**Current Smoker with Moderate Pack Years of Smoking (20 - 40 pack years of smoking)**	30757	1015	31906	1038
	**Current Smoker with Light Pack Years of Smoking (0 - 20 pack years of smoking)**	26775	453	20259	429

[a]Breast, colorectal, lung, or prostate Cancer in first-degree relatives (nonadopted father, mother, siblings)

Supplementary Table 3. Descriptive Statistics of the UK Biobank Study Population.

Cancer Type	ICD-10 Code	Incident Cancer Cases (Female)	Incident Cancer Cases (Male)	Number of SNPs included in each PRS
**Bladder**	C67	57	230	12
**Breast**	C50	2855	-	306
**Colorectum**	C18-C20	681	957	67
**Endometrium**	C54, C55	477	-	16
**Kidney**	C64	127	249	12
**Lung**	C33-C34	494	554	43
**Malignant Melanoma**	C43	408	386	65
**Non-Hodgkin Lymphoma**	C82, C83.0, C83.3, C83.8, C85.2, C88.4	277	326	20
**Ovary**	C56	282	-	25
**Pancreas**	C25	149	182	19
**Prostate**	C61	-	3022	125
**Total**		5807	5906	

Supplementary Table 4. Cancer types with the corresponding ICD-10 codes, incident cancer cases, and the number of SNPs included in each cancer PRS.

Supplementary References

1. Lennon AM, Buchanan AH, Kinde I, Warren A, Honushefsky A, Cohain AT, et al. Feasibility of blood testing combined with PET-CT to screen for cancer and guide intervention. Science. 2020;369:eabb9601.

2. Klein, E. A. *et al.* Clinical validation of a targeted methylation-based multi-cancer early detection test using an independent validation set. *Ann. Oncol. Off. J. Eur. Soc. Med. Oncol.* **32**, 1167–1177 (2021).
