# Supplementary material for: Potential utility of risk stratification for multicancer screening with liquid biopsy tests
Source: NPJ Precis Oncol. 2023 Apr 22;7:39. doi: 10.1038/s41698-023-00377-w (PMC10122653; doi:10.1038/s41698-023-00377-w)
Supplement: Supplementary file 3 — REPORTING SUMMARY [file 41698_2023_377_MOESM3_ESM.pdf]

nature portfolio

Corresponding author(s): Nilanjan Chatterjee

Last updated by author(s): Mar 14, 2023

Reporting Summary

Nature Portfolio wishes to improve the reproducibility of the work that we publish. This form provides structure for consistency and transparency in reporting. For further information on Nature Portfolio policies, see our Editorial Policies and the Editorial Policy Checklist.

Statistics

For all statistical analyses, confirm that the following items are present in the figure legend, table legend, main text, or Methods section.

n/a	Confirmed	
☐	☒	The exact sample size (*n*) for each experimental group/condition, given as a discrete number and unit of measurement
☐	☒	A statement on whether measurements were taken from distinct samples or whether the same sample was measured repeatedly
☐	☒	The statistical test(s) used AND whether they are one- or two-sided<br>*Only common tests should be described solely by name; describe more complex techniques in the Methods section.*
☐	☒	A description of all covariates tested
☐	☒	A description of any assumptions or corrections, such as tests of normality and adjustment for multiple comparisons
☐	☒	A full description of the statistical parameters including central tendency (e.g. means) or other basic estimates (e.g. regression coefficient) AND variation (e.g. standard deviation) or associated estimates of uncertainty (e.g. confidence intervals)
☐	☒	For null hypothesis testing, the test statistic (e.g. *F*, *t*, *r*) with confidence intervals, effect sizes, degrees of freedom and *P* value noted<br>*Give P values as exact values whenever suitable.*
☒	☐	For Bayesian analysis, information on the choice of priors and Markov chain Monte Carlo settings
☒	☐	For hierarchical and complex designs, identification of the appropriate level for tests and full reporting of outcomes
☐	☒	Estimates of effect sizes (e.g. Cohen's *d*, Pearson's *r*), indicating how they were calculated

Our web collection on statistics for biologists contains articles on many of the points above.

Software and code

Policy information about availability of computer code

Data collection	Not applicable. The study involves analysis of existing data.
Data analysis	This project was developed using R version 4.2.2. and the codes used for the project are available from Github at https://github.com/eswk-im/PCRSanalysis-.git.

For manuscripts utilizing custom algorithms or software that are central to the research but not yet described in published literature, software must be made available to editors and reviewers. We strongly encourage code deposition in a community repository (e.g. GitHub). See the Nature Portfolio guidelines for submitting code & software for further information.

Data

Policy information about availability of data

All manuscripts must include a data availability statement. This statement should provide the following information, where applicable:

- Accession codes, unique identifiers, or web links for publicly available datasets
- A description of any restrictions on data availability
- For clinical datasets or third party data, please ensure that the statement adheres to our policy

UK Biobank data are available through application to the UK Biobank Access Management System (AMS), https://www.ukbiobank.ac.uk/enable-your-research/register. GWAS summary statistics of the single-nucleotide polymorphisms (SNPs) used for polygenic risk score (PRS) construction for each trait is available in the supplementary information section with relevant source literatures.

Human research participants

Policy information about studies involving human research participants and Sex and Gender in Research.

Reporting on sex and gender	We utilized both self-reported sex (UK Biobank Data Showcase Data-Field 31) and genetic sex (UK Biobank Data Showcase Data-Field 22001). Participants with self-reported sex and genetic sex mismatch were excluded from analysis. We developed a sex-specific pan-cancer risk prediction model to estimate the risk of developing at least one cancer over the course of follow-up. The multicancer model included eleven cancer types (bladder, breast [Female-only], colorectum, endometrium [Female-only], kidney, lung, melanoma, Non-Hodgkin's lymphoma, ovary [Female-only], pancreas, and prostate [Male-only]). Quality control procedures resulted in a study population involving 133,830 unrelated females and 115,207 unrelated males of White British ancestry.
Population characteristics	The study involves analysis of existing data and does not involve new human research participants. Descriptive statistics of the UK Biobank participants included in our analysis are available in the supplementary information section.
Recruitment	UK Biobank is a prospective epidemiological cohort study with over 500,000 participants. Individuals aged 40-69 at baseline were recruited across the United Kingdom from 2006-2010. More information on participant recruitment available at Bycroft, C., Freeman, C., Petkova, D. et al. The UK Biobank resource with deep phenotyping and genomic data. Nature 562, 203–209 (2018). https://doi.org/10.1038/s41586-018-0579-z.
Ethics oversight	Please refer to https://www.ukbiobank.ac.uk/learn-more-about-uk-biobank/about-us/ethics for detailed information on UK Biobank research ethics approval.

Note that full information on the approval of the study protocol must also be provided in the manuscript.

Field-specific reporting

Please select the one below that is the best fit for your research. If you are not sure, read the appropriate sections before making your selection.

☒ Life sciences ☐ Behavioural & social sciences ☐ Ecological, evolutionary & environmental sciences

For a reference copy of the document with all sections, see nature.com/documents/nr-reporting-summary-flat.pdf

Life sciences study design

All studies must disclose on these points even when the disclosure is negative.

Sample size	We studied involved 133,830 female and 115,207 unrelated male participants of White British ancestry aged 40-73, with 5,807 and 5,906 incident cancer cases, respectively, over the course of follow-up. No statistical method was used to predetermine sample size. Maximal dataset was retained for analysis after data exclusions.
Data exclusions	Prior to data analysis, we excluded individuals who were lost to follow-up, with genetic sex and self-reported sex mismatch, those with any cancer diagnosis prior to baseline assessment (prevalent cancers), and participants with missing data in any one of the classical risk factors (BMI, smoking status, pack years of smoking, and family history of cancer in non-adoptive first-degree relatives). Participants without imputed genotype data were excluded from the analysis. These quality control procedures resulted in a study population involving 133,830 unrelated females and 115,207 unrelated males of White British ancestry.
Replication	Our UK Biobank dataset was split into training and test set. We trained and validated a Cox proportional hazards model that allows estimation of absolute risk of developing at least one of the eleven cancers included in our study for females and males, separately. Results can be replicated by using the code provided above. Additional codes and information can be provided by request. Codes were reviewed thoroughly. Results were replicated successfully.
Randomization	Not an experimental study. Randomization was not relevant.
Blinding	Blinding was not relevant.

Reporting for specific materials, systems and methods

We require information from authors about some types of materials, experimental systems and methods used in many studies. Here, indicate whether each material, system or method listed is relevant to your study. If you are not sure if a list item applies to your research, read the appropriate section before selecting a response.

Materials & experimental systems

n/a	Involved in the study
☒	☐ Antibodies
☒	☐ Eukaryotic cell lines
☒	☐ Palaeontology and archaeology
☒	☐ Animals and other organisms
☒	☐ Clinical data
☒	☐ Dual use research of concern

Methods

n/a	Involved in the study
☒	☐ ChIP-seq
☒	☐ Flow cytometry
☒	☐ MRI-based neuroimaging
